# Efficacy of a quadruple therapy regimen for *Helicobacter pylori* eradication after partial gastrectomy

**DOI:** 10.1590/1414-431X20155080

**Published:** 2016-02-05

**Authors:** F. Zhang, Z.J. Bao, D.M. Shi, P. Xiang, L. Xiao, Y.Q. Huang, G.S. Zhang, S.M. Yin

**Affiliations:** Division of Gastroenterology, Huadong Hospital, Shanghai Medical College, Fudan University, Shanghai, China

**Keywords:** Helicobacter pylori, Gastrectomy, Gastric stump, Eradication, Efficacy

## Abstract

We aimed to evaluate the effectiveness and safety of bismuth-containing quadruple therapy plus postural change after dosing for *Helicobacter pylori* eradication in gastrectomized patients. We compared 76 gastric stump patients with *H. pylori* infection (GS group) with 50 non-gastrectomized *H. pylori*-positive patients who met the treatment indication (controls). The GS group was divided into GS group 1 and GS group 2. All groups were administered bismuth potassium citrate (220 mg), esomeprazole (20 mg), amoxicillin (1.0 g), and furazolidone (100 mg) twice daily for 14 days. GS group 1 maintained a left lateral horizontal position for 30 min after dosing. *H. pylori* was detected using rapid urease testing and histologic examination of gastric mucosa before and 3 months after therapy. Mucosal histologic manifestations were evaluated using visual analog scales of the updated Sydney System. GS group 1 had a higher prevalence of eradication than the GS group 2 (intention-to-treat [ITT]: P=0.025; per-protocol [PP]: P=0.030), and the control group had a similar prevalence. GS group 2 had a lower prevalence of eradication than controls (ITT: P=0.006; PP: P=0.626). Scores for chronic inflammation and activity declined significantly (P<0.001) 3 months after treatment, whereas those for atrophy and intestinal metaplasia showed no significant change. Prevalence of adverse reactions was similar among groups during therapy (P=0.939). A bismuth-containing quadruple therapy regimen plus postural change after dosing appears to be a relatively safe, effective, economical, and practical method for *H. pylori* eradication in gastrectomized patients.

## Introduction

*Helicobacter pylori* infection is associated with chronic gastritis, peptic ulceration, gastric carcinoma, and malignant gastric lymphoma (1 2 3). However, it is also a factor for metachronous gastric cancer after surgery for early gastric cancer (4). *H. pylori* colonizes mainly the bottom of the gastric mucus layer and surface of epithelial cells. It may disappear spontaneously after partial gastrectomy, but can cause reinfection *via* fecal-oral, gastric-oral, and oral-oral routes (5,6).

Some researchers believe that a gastric remnant does not increase the risk of cancer (7), and that eradication of *H. pylori* in gastrectomized patients is not necessary (8). However, most researchers consider a remnant stomach to be a special pre-cancerous condition, and that treatment of *H. pylori* infection in patients with a residual stomach is as important as treatment of *H. pylori* infection in the general population (9 10 11 12).

Routine treatment for *H. pylori* is established and has been used in many countries (1,). However, the pH, anatomy, gastric motility, and distribution of *H. pylori* are altered after partial gastrectomy, which can adversely affect use of anti-*H. pylori* drugs. Consensus on the therapeutic regimen and efficacy evaluation for *H. pylori* eradication in a remnant stomach is lacking (16). Only a few authors have reported eradication therapy of *H. pylori* in the residual stomach, and most have used classical proton pump inhibitor (PPI)-based triple therapy. Different PPIs have been used in different studies, but the major antibiotics administered have been amoxicillin and clarithromycin for 1–4 weeks. Prevalence of eradication per-protocol (PP) based on omeprazole, lansoprazole, and rabeprazole has been reported to be 42.1-84.6% (17,18), 44-90% (11,), and 83.1-90.9% (23,24), respectively. With regard to the dosing method, one study showed that triple therapy plus postural change can improve the prevalence of eradication of *H. pylori* in patients after partial gastrectomy (19 [Bibr B20]). Possibly because of the availability and safety of bismuth, bismuth-containing quadruple therapy is seldom recommended in *H. pylori*-positive gastrectomized patients. Antibiotic resistance of *H. pylori* in China is relatively high, so a short course of a bismuth-containing quadruple therapy regimen (1 or 2 weeks) has received attention (14 [Bibr B15]).

We used standardized bismuth-containing quadruple therapy plus postural change after drug ingestion to treat *H. pylori* infection in patients with a gastric stump. We evaluated the effectiveness and safety of this regimen to provide a suitable method for *H. pylori* eradication in patients with a gastric stump.

## Subjects and Methods

The study protocol was approved by the Ethics Committee of Huadong Hospital (affiliated to Fudan University, Shanghai, China). Written informed consent was obtained from each individual involved in the study. In total, 76 *H. pylori*-positive patients who had undergone partial gastrectomy in Huadong Hospital were enrolled in the gastric pump (GS) group according to the following inclusion criteria: duration after subtotal gastrectomy was ≥1 year; distal gastrectomy with Billroth I (B-I) or Billroth II (B-II) anastomosis; surgical indication was benign peptic ulceration or early gastric cancer; no chemotherapy, radiotherapy, or other surgery was undertaken <6 months before study start. All 76 patients were then allocated to GS group 1 (38 patients) and GS group 2 (38 patients) non-randomly to make sure that the baseline conditions (including age, sex, indication for gastrectomy, reconstruction method, and duration after surgery) of the two groups were equal to each other. Contemporary *H. pylori*-positive non-gastrectomized patients diagnosed with chronic gastritis who had dyspepsia symptoms and met the treatment indication were allocated to the control group (50 patients). Ages and sexes of the control group were matched with those of the GS group.

Exclusion criteria were: history of *H. pylori* treatment; treatment with antibiotics, PPIs, H_2_-receptor antagonists, bismuth salts, or traditional Chinese medicines <1 month before study start; contraindications to endoscopic examination or histologic tests; allergy to any drug used in the present study; history of hemorrhage, obstruction, perforation, or other complications within the digestive system.

### Therapeutic regimen for *H. pylori* eradication

Quadruple therapy involving bismuth was administered to GS and control groups. Medications were bismuth potassium citrate (220 mg), esomeprazole (20 mg), amoxicillin (1.0 g), and furazolidone (100 mg): this quadruple therapy regimen was abbreviated to BEAF. Each medication was administered twice daily for 14 days. Patients in GS group 1 were required to maintain the left lateral horizontal position for 30 min after each dose.

### Endoscopy and gastric mucosal biopsy

Gastroscopy was undertaken before and 3 months after eradication therapy (3 months after medication was stopped). Biopsy specimens from six sites were obtained for rapid urease testing (RUT). Histologic examination was done using Giemsa staining. Of these, two-each were from the lesser and greater curvature of the middle-high corpus, and two-each from the gastric side of the stoma or antral lesser curvature, in that order. Histopathologic findings (chronic inflammation, activity, atrophy, intestinal metaplasia) based on the visual analog scale of the updated Sydney System (25) were graded (none = 0, mild = 1, moderate = 2, severe = 3). Mean scores of the three sites of each subject were recorded. All histopathologic diagnoses were completed by an experienced pathologist blinded to clinical information.

### Diagnostic criteria for infection and eradication of *H. pylori* (14)

Before eradication therapy, *H. pylori* infection was confirmed if RUT or histologic examination was positive. Three months after treatment, eradication was deemed to be successful if both tests were negative, or to have failed if either was positive.

### Cancer staging systems

The American Joint Committee on Cancer Cancer Staging Manual (7th edition) (26) was used for cancer staging.

### Statistical analyses

Measurement data are reported as the mean±SD. Comparisons between groups were made using the Student's *t*-test. Comparison among multiple groups was done by one-way analysis of variance.

Categorical data are reported as percentages. Comparison among groups was done using the *χ^2^* test. The effect of different surgical procedures was analyzed by the *χ* ^2^_MH_ test. *H. pylori* eradication was analyzed by intention-to-treat (ITT) and PP. P<0.05 was considered significant.

## Results

Seventy-six gastrectomized and 50 non-gastrectomized patients with *H. pylori* infection were enrolled. No significance was found for age or sex among the three groups (P>0.05). Differences in indications for gastrectomy, reconstruction method, and duration after surgery of the two GS groups were not significant (P>0.05). Clinical data of GS and control groups are shown in Table 1.

**Table:**
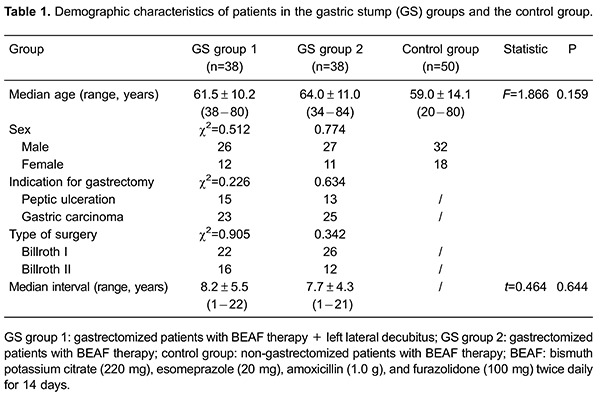

A total of 73 GS and 48 control-group subjects completed 2 weeks of treatment. Five patients (one from the GS group 1; 2 from the GS group 2; two from the control group) dropped out of the study because of the side effects of quadruple therapy. Three months after treatment, the prevalence of eradication was significantly higher in GS group 1 than in GS group 2 according to ITT and PP analyses (ITT: *χ^2^=*5.050, P*=*0.025; PP: *χ^2^=*4.715, P*=*0.030). Prevalence of eradication was similar in GS group 1 and the control group, but the difference was not significant (ITT: *χ^2^=*0.090, P*=*0.765; PP: *χ^2^=*0.238, P*=*0.626). Prevalence of eradication in GS group 2 was lower than that in the control group based on the two analyses, and the difference was significant (ITT: *χ^2^=*7.418, P*=*0.006; PP: *χ^2^=*7.897, P*=*0.005). Prevalence of eradication in the three groups is shown in Table 2.

**Table:**
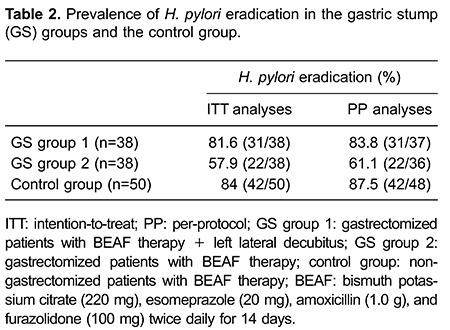

The Mantel-Haenszel (MH) test was done to compare the prevalence of eradication for B-I and B-II in GS group 1 and GS group 2 by ITT and PP: the difference was not significant (ITT: *χ* ^2^_MH_=0.072, P*=*0.789, odds ratio MH [*OR*_MH_]=0.741; PP: *χ* ^2^_MH_=0.044, P*=*0.833, *OR*_MH=_0.748). This observation suggested that the surgical method (B-I or B-II) did not affect the prevalence of eradication of *H. pylori* if the effect of different regimens (postures) was not considered. Prevalence of eradication for different surgical methods by GS group is shown in Table 3.

**Table:**
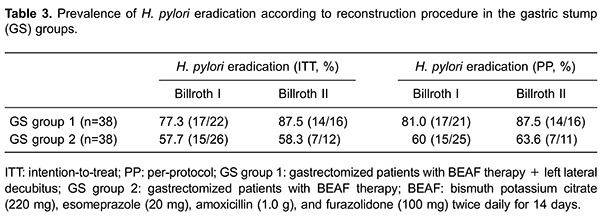

**Figure 1:**
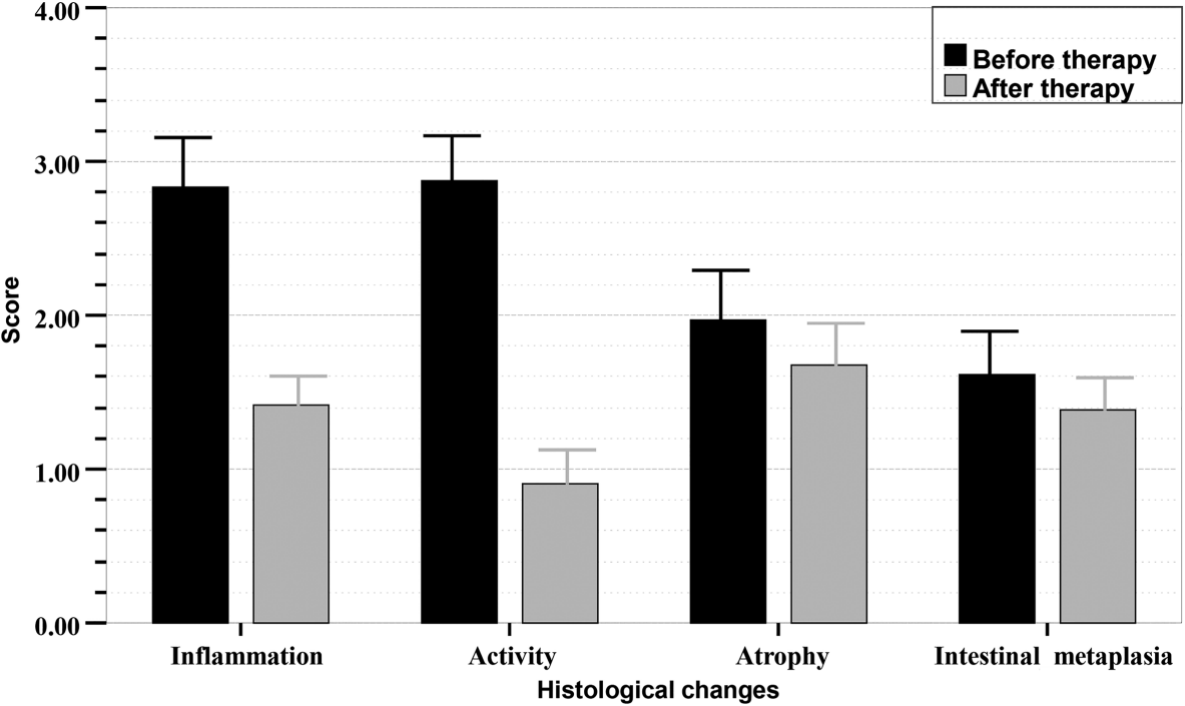
Sequential histologic mucosal changes in gastric stump (GS) group 1 (gastrectomized patients with BEAF therapy + left lateral decubitus) before and after *Helicobacter pylori* eradication therapy, scored according to the updated Sydney system. BEAF therapy was bismuth potassium citrate (220 mg), esomeprazole (20 mg), amoxicillin (1.0 g), and furazolidone (100 mg) twice daily for 14 days. Scores for inflammation and activity in GS group 1 with successful *H. pylori* eradication therapy decreased significantly (P<0.001, Student's *t*-test). Scores for atrophy and intestinal metaplasia in this group were not significantly different after *H. pylori* treatment (P>0.05).

Of the patients who completed the study (31 from the GS group 1; 22 from the GS group 2; 42 from the control group), the scores for chronic inflammation and activity decreased remarkably 3 months after eradication therapy, and the difference was significant (P<0.001). However, the scores for atrophy and intestinal metaplasia did not change markedly, and the difference was not significant (P>0.05). Histologic scores of the gastric mucosa in the three groups before and after treatment are shown in Figures 1-3.

**Figure 2 f02:**
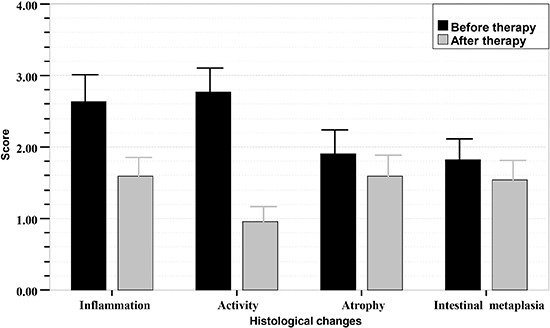
Sequential histologic mucosal changes in gastric stump (GS) group 2 (gastrectomized patients with BEAF therapy) before and after *H. pylori* eradication therapy, scored according to the updated Sydney system. BEAF therapy was bismuth potassium citrate (220 mg), esomeprazole (20 mg), amoxicillin (1.0 g), and furazolidone (100 mg) twice daily for 14 days. Scores for inflammation and activity in the group with successful *H. pylori* eradication therapy decreased significantly (P<0.001, Student's *t*- test). Scores for atrophy and intestinal metaplasia in this group did not change significantly after *H. pylori* treatment (P>0.05).

**Figure 3:**
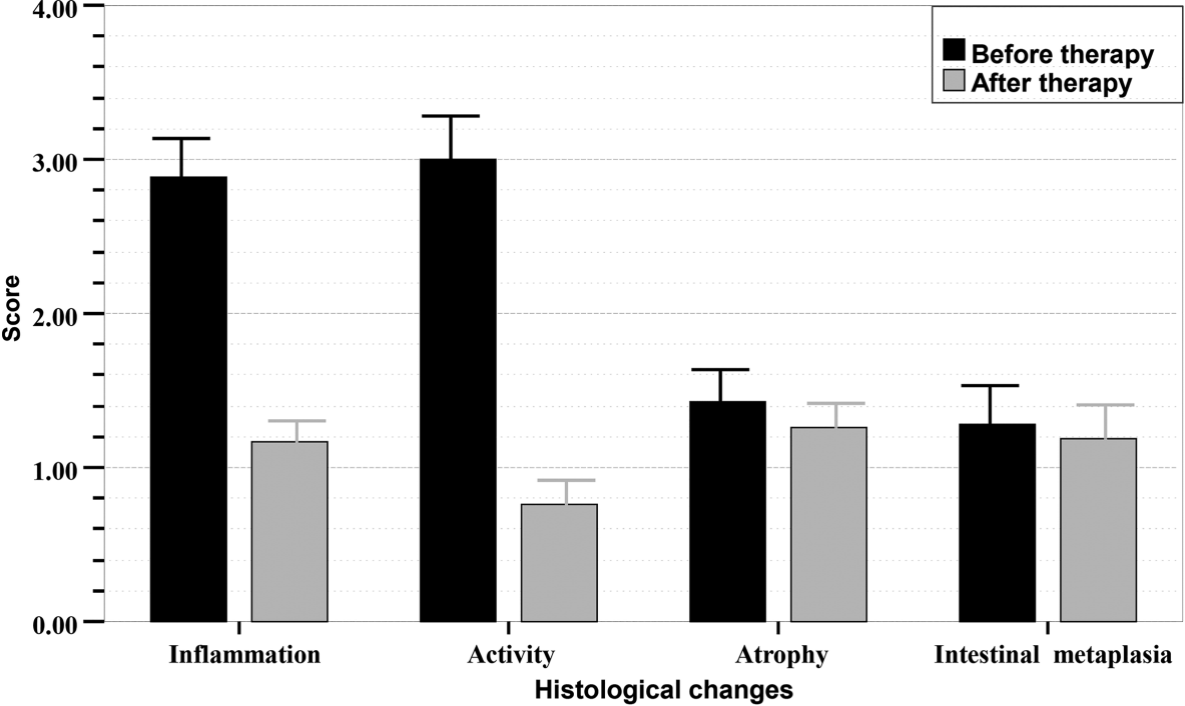
Sequential histologic mucosal changes in the control group (non-gastrectomized patients with BEAF therapy) before and after *H. pylori* eradication therapy, scored according to the updated Sydney system. BEAF therapy was bismuth potassium citrate (220 mg), esomeprazole (20 mg), amoxicillin (1.0 g), and furazolidone (100 mg) twice daily for 14 days. Scores for inflammation and activity in the group with successful *H. pylori* eradication therapy decreased significantly (P<0.001, Student's *t-*test). Scores for atrophy and intestinal metaplasia scores in the group did not change significantly after *H. pylori* treatment (P>0.05).

Adverse events that occurred during treatment are listed in Table 4. One case of “headache and dizziness” was reported in GS group 1. One case of “nausea and vomiting” and one of “diarrhea” was documented in GS group 2. One case of nausea and vomiting, and one of headache and dizziness, was documented in the control group. These five patients dropped out of the study.

**Table:**
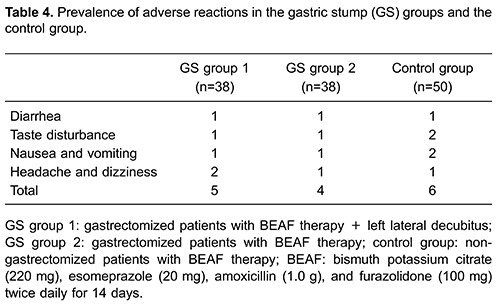

Other side effects were tolerable and disappeared gradually after treatment. Severe adverse events were not observed. Prevalence of adverse reactions in GS group 1, GS group 2, and the control group was 13.2% (5/38), 10.5% (4/38), and 12% (6/50), respectively. No significant difference was detected among groups.

## Discussion

Eradication has become first-line treatment for *H. pylori*-associated diseases (4,13). However, a standardized protocol for patients with a gastric stump has not been established. Conventional therapy for patients with an intact stomach has been applied to gastric stump patients in most studies (11,19,21), but is not as effective as in the general population. This inferior efficacy may be associated with the baseline conditions (age, sex, surgical procedure, disease history) of study populations; the species, dose, treatment course, and drug resistance for PPIs and antibiotics may also be influential factors. Surgical methods and therapeutic regimens for gastric-remnant patients can also create significant differences. We found no significant differences in demographic or surgical characteristics among groups. Time elapsed between gastrectomy and eradication treatment was >1 year to avoid the effect of preoperative and postoperative treatments (antibiotics, chemotherapy). Only two surgical methods (B-I, B-II) were included in this pilot study; we did not consider lymph node dissection or other procedures.

Clinical and animal studies (27,28) have shown that eradication of *H. pylori* is reliant upon sufficient and effective concentrations of antimicrobial drugs in the gastric mucosa. Drugs administered *via* the oral route can be delivered to the stomach directly (drugs enter the gastric lumen *via* oral administration) or indirectly (drugs are absorbed into the circulation in the intestine, and are excreted into the gastric lumen by gastric epithelial cells); both routes are equally important for *H. pylori* eradication. After partial gastrectomy, gastric emptying may be accelerated secondary to dissection of the gastric antrum. Thus, drugs administered *via* the oral route may not be topically effective (direct delivery) because the retention time is too short. Moreover, the time for absorption in the proximal intestine is reduced, and ingested drugs cannot attain an effective systemic concentration (indirect delivery). Reducing direct and indirect delivery methods leads to insufficient concentrations of antimicrobial drugs in the remnant stomach, which affects treatment efficacy. Simultaneously, the action of PPIs (enteric-coated tablets), which are mainly delivered indirectly, is also impeded (28). Additional factors that affect direct delivery (forms of drug dose, gastric mucus barrier) and indirect delivery (residual area of the gastric mucosa, gradient of drug concentration, permeability of gastric epithelial cells), as well as extension of lymph node dissection could also influence eradication of *H. pylori* after gastrectomy (19). Thus, exploration and establishment of a suitable and effective therapeutic regimen for *H. pylori* eradication in patients with a residual stomach is important.

As a country with high resistance to antimicrobial agents, only one of the five regimens recommended by Maastricht-IV is suitable in China: bismuth-containing quadruple therapy. This therapy has been shown to increase the eradication rate by 8–14% (14). We chose amoxicillin and furazolidone, which were inexpensive and have a lower prevalence of antimicrobial resistance. Results showed that the prevalence of eradication with BEAF was lower in gastrectomized patients (GS group 2) than in non-gastrectomized patients (control group) according to ITT and PP analyses. This finding suggested that the anatomic and functional alteration of the gastrectomized stomach could impair the efficacy of routine therapy for *H. pylori* eradication. This result was consistent with the results of a study by Kim et al. (23).

According to Kato et al. (12), the prevalence of eradication in remnant-stomach patients correlates positively with the pH gradient of gastric juice to the same extent as that seen in patients with *H. pylori* infection. To increase the chances of eradication, we used esomeprazole to inhibit secretion of gastric acid, elevate gastric pH, as well as to improve the stability and permeability of antibiotics.

*H. pylori* infection is distributed focally in the stomach. With the changes in the stomach environment after surgery, infection-susceptible areas migrate to the proximal residual stomach (gastric corpus and fundus) (6,29). With reflux of alkaline digestive juices (more significantly in B-II than in B-I), the pH in the gastric lumen increases, which is not conducive for the survival of *H. pylori*. This phenomenon renders a lower prevalence of *H. pylori* infection postoperatively (5,9) (lower in B-II than in B-I (16,23)). The research of Honda et al. (22) and Kim et al. (23) suggests that the effect of eradication therapy does not differ whether given preoperatively or postoperatively because postoperative physiologic changes did not have an adverse impact on *H. pylori* eradication. They found that the reconstruction method did not affect the efficacy of eradication in the gastric remnant, a finding that was in accordance with our study. This observation could be because the: (i) 14-day eradication therapy with BEAF was so effective that the impact of different reconstruction methods was not evident; (ii) duration after surgery and degree of reflux of alkaline digestive juice varied, thereby obscuring the effect of different surgical procedures; (iii) sample size of our study was too small to reveal the effect of surgical methods.

Cooreman et al. (30) found that the local gastric concentration of antimicrobial drugs was higher than that of serum 15 min after dosing, and peaked at 30 min. Upon application of an optimized ^13^C-urea breath test (UBT) in patients after partial gastrectomy (31 32 33), we asked gastrectomized patients to maintain a left lateral horizontal position for 30 min after dosing to prolong the retention time of the drug in the stomach, thereby enhancing direct and indirect delivery and increasing the chance of eradication. We showed that the prevalence of eradication of BEAF plus postural alteration in gastrectomized patients was similar to that in non-gastrectomized patients, and higher than that obtained with BEAF alone in gastrectomized patients. These findings suggested that BEAF combined with postural alteration had a good effect on *H. pylori* eradication in gastrectomized patients. For efficacy, and to avoid the inconvenience of postural changes, we recommend medication dosing before arising in the morning and before bedtime.

The mechanism of action of gastric stump cancer is similar to that of primary gastric cancer: a complicated, multifactorial process, with its own unique features. *H. pylori*-infected gastric-remnant mucosa eventually progresses to gastric cancer after the following steps: chronic gastritis, atrophy, intestinal metaplasia, and dysplasia (4,34). Studies have shown that eradication of *H. pylori* can inhibit the progress of gastric mucosal atrophy and intestinal metaplasia in the intact stomach (1,35,36) and gastric remnant (21,37), which reduces the risk of carcinogenesis. In consideration of the recurrence risk of 9.6–13.5% (38) and differences in compliance of gastrectomized patients for retesting by gastroscopy and ^13^C-UBT, evaluation of the endpoint was 3 months after treatment. We showed that the scores for atrophy and intestinal metaplasia of patients in the GS group did not change significantly 3 months after *H. pylori* eradication therapy. However, longer follow-up for histologic changes of the lesser curvature, great curvature, and lesser-curvature mucosa might be needed to obtain a reliable conclusion (35,37). However, inflammation and activity improved greatly, which was consistent with the study by Hamaguchi et al. (21). In addition, the prevalence of adverse events was similar in GS and control groups. The economic burden did not increase in gastric stump patients, demonstrating that BEAF plus postural change has good efficacy and is safe for *H. pylori* eradication in gastric-remnant patients.

In conclusion, BEAF plus postural change (left lateral horizontal position) is a relatively safe, effective, economical, and practical method for *H. pylori* eradication in gastrectomized patients. The efficacy and safety of other standardized therapies for *H. pylori* eradication plus postural change in gastric-remnant patients merits further research.
